# Waiting times for health services, health, and labour market outcomes

**DOI:** 10.1093/eurpub/ckaf213

**Published:** 2025-12-02

**Authors:** Luigi Siciliani

**Affiliations:** Department of Economics and Related Studies, University of York, York, United Kingdom

## Abstract

Waiting times for health care is a significant health policy concern across many health systems, which has been exacerbated by the COVID-19 pandemic. Long waiting times for non-emergency care generate health losses to patients because health benefits are postponed. They can increase the risk of mortality or morbidity and reduce patient ability to benefit from health care. Waiting times can also generate negative spill-over effects on labour market outcomes. For individuals in the working age, employed individuals might end up on sick leave and claim sickness benefits, or experience reduced productivity if they continue to work. Individuals looking for a job may find it harder to find employment or become economically inactive. We conduct a narrative review of the literature on the effect of waiting times on health losses and labour market outcomes. There is growing literature documenting the effect of longer waiting times on labour market outcomes. Although limited, the literature identifies potentially harmful effects in particular when patients are waiting for mental health services and orthopaedic treatment. The findings have implications for prioritization of patients on the list and for allocation of resources within the health sector and across sectors.

## Introduction

Waiting times for non-emergency health care are a significant health policy concern across many health systems where patients must wait weeks or months to access health services [1]. Waiting times differ extensively across countries as a result of different capacity constraints, funding decisions, health personnel, and mismatch with growing demand for health care driven by an ageing of the population and technological innovation. In 2022, the median waiting time for a hip replacement, a common planned surgical procedure, was 74 days in Spain, 92 days in Ireland, 147 days in Portugal, and 525 days in Slovenia. The proportion of patients waiting more than 3 months between addition to the waiting list and treatment was between 40% and 50% in Denmark, Sweden, Finland and Estonia, between 60% and 70% in Portugal, Costa Rica, Australia, and Spain, and between 70% and 80% in New Zealand and Norway. These examples highlight how waiting times for non-emergency care are a significant health policy issue across a range of institutional arrangements on both funding and provision [2].

Waiting time is also a significant source of unmet need for medical care. In 2023, being on a waiting list was a primary reason for unmet need in Estonia, Finland, Latvia, Lithuania, Slovenia, Poland, Denmark, Ireland, Sweden, Spain, Hungary, Austria, Netherlands (waiting list accounted for more than 50% of unmet need due to care being too expensive, being on waiting list, or too far to travel [3]).

COVID-19 disrupted non-emergency care because health systems had to suspend many treatments to divert efforts towards COVID-19 patients and avoid patients being infected while seeking care. Elective care was suspended for 4–12 weeks in the first wave of the pandemic, and to a lower extent in subsequent waves. Surgical procedures reduced by 18% on average across 31 OECD countries between 2019 and 2020 for a bundle of 15 common surgical procedures, which led to a marked increase in waiting times [4]. Reductions in elective care during the first year were followed by a partial recovery in the second year. Such disruptions have generated backlogs. Waiting lists and waiting times have further increased as a result [4, 5]. Relative to 2019, the waiting time of the patient on the list for six common elective surgeries (hip and knee replacement, cataract surgery, prostatectomy, coronary bypass, and angioplasty) across nine OECD countries increased by about 27%–30% in the first 3 consecutive years, and 16% in the fourth year. This is against a reduction in volume for the same procedures of about 19% and 10% in the first- and second-year following COVID-19, before returning to similar levels in the pre-pandemic period [6].

In this article, we review the existing evidence that quantifies the health losses generated by longer waiting times for non-emergency care that could arise from postponing health benefits, health deterioration while waiting, reduced ability to benefit from health, in addition to increased mortality risk and morbidity. We also review the limited but growing evidence which quantifies the effect of longer waiting times on labour market outcomes, for example, if patients waiting for health care end up on sick leave, continue working with reduced productivity or become inactive [7, 8].

It has long been recognized that collaboration between sectors is needed because health is affected by other sectors (e.g. transport policies reduce road injuries and deaths, environmental policies can reduce pollution and improve health, and education policies can induce healthier lifestyle) and other sectors are affected by health or health systems (e.g. better health can improve education attainment, labour market outcomes, and reduce poverty). This idea is encompassed by the Health in All Policies approach which suggests that all sectors should work together [9]. Health can generate co-benefits for other sectors and other sectors can generate co-benefits for health or health systems.

In this study, we focus specifically on the co-benefits that could arise by reducing waiting times in health care. It highlights possible co-benefits from health (SDG3) to employment (SDGs 9.5, 0.6) and from the health sector to labour markets [9–11]. Although longer waiting times themselves can generate a cost, as opposed to a co-benefit, to other domains, the reduction in waiting times through health system interventions can generate co-benefits on labour market outcomes, in addition to improving health.

The focus of this study is on waiting times for non-emergency care, which is generally measured from the time a specialist adds a patient on the waiting list until the patient receives treatment or surgery (though some countries measure waiting times from GP referral to treatment to better match with the patient pathway). We do not focus on emergency care when patients wait in the Accident and Emergency department at the hospital which raises related but distinct issues as patients queue at the hospital site as opposed to being added to the list and come back later for treatment [1].

## Methods

We conducted a narrative review of the existing academic literature on the effect of waiting times on health losses and labour market outcomes. Given that the literature from waiting times on labour market outcome is recent and limited, we also briefly refer to other review studies that document the effect of health or health policies on labour market outcomes. The literature review search was conducted between April and July 2025. Literature was drawn from ScienceDirect, Google Scholar and PubMed. Search terms included: waiting times, waiting lists, backlogs, health care, health, employment, sickness benefits, labour market outcomes.

We include only studies published after 2005, and that use large representative samples of patient population (from administrative data that aim to capture the whole population of patients with a given condition, treatment or area of care). We also include only studies that have a robust statistical design (through the control of an extensive range of variables or causal identification, e.g. through an instrumental variable approach). We include both peer-reviewed journal articles and working papers that are in the public domain.

## Results

A total of 20 empirical studies were included. The studies used data from the United Kingdom (*n* = 11), Norway (*n* = 4), Australia (*n* = 2), the Netherlands (*n* = 1), Korea (*n* = 1), and the US (*n* = 1). Four studies focused on waiting times and labour outcomes. Three studies focused on mental health services. Seventeen studies were published in peer-reviewed journals.

### Health losses arising from waiting

Waiting times for non-emergency care generate health losses to patients while waiting because health benefits are postponed [12]. In addition, health may deteriorate while waiting and waiting times could reduce patient ability to benefit from health care once the treatment is eventually received [13]. In more dramatic cases, a longer wait can increase the risk of mortality or morbidity. For example, patients with cardiovascular conditions can experience worsening symptoms and an increase in the probability of preoperative death and unplanned emergency admission [14].

Several studies have tested the effect of waiting times on health outcomes. Nikolova *et al*. [15] found that long waits in England reduce health-related quality of life for hip and knee replacement patients, as measured by patient-reported outcome measure, but no effect was found for varicose veins and inguinal hernia. Moscelli *et al*. [16] found no evidence that waiting times in England are associated with higher in-hospital mortality for coronary bypass but they found an association between waiting times and emergency readmission following a surgery. Arabadzhyan *et al*. [17] found that, in the post COVID-19 years, longer waiting times for coronary artery bypass grafting increased mortality and length of stay, but no effect was found for percutaneous transluminal coronary angioplasty neither before or after COVID-19. Godøy *et al*. [7] showed that in Norway long waiting times for orthopaedic surgery did not increase healthcare utilization (e.g. due to worsened health status) or health outcomes (e.g. mortality, emergency readmissions).

Costantini [18] found that longer waiting time for mental health care for veterans in the US increased mortality. This is because longer waiting times increase the probability that patients miss their follow-up mental health visit, and permanently disengage from care. Reichert and Jacobs [19] found that individuals with longer waiting times for psychosis services in England are significantly associated with a deterioration in patient outcomes 12 months after acceptance for treatment for psychosis. The outcomes were measured on the Health of the Nation Outcome Scale, which is designed to assess the health and social functioning of people and is reported by clinicians. The effects were strongest for waiting times longer than 3 months. For cancer, Han *et al*. [20] showed that in South Korea patients with lung cancer receiving surgery after 30 days are associated with an increased mortality rate of 15%.

Even if waiting times do not affect the ability to benefit from health care, there is still a health loss caused by the postponement of the health benefit. The health loss per unit of time is larger the more effective is the treatment in improving health. Gibbs *et al*. [21, 22] ran a simulation study to estimate the health loss arising from an additional 6 weeks of waiting, measured in Quality-Adjusted Life Years (QALYs), for a selection of high-volume elective surgeries in England. They find that the largest health loss from waiting is for hip and knee replacement, followed by hysterectomy, hernia, cholecystectomy, cataract, coronary bypass, and percutaneous coronary intervention.

One way to minimize the total loss from waiting is to enhance waiting time prioritization, which involves reducing waiting times for patients with higher urgency, need and severity and increasing waiting times for those with lower urgency, need and severity [23]. There is evidence of prioritization across treatments. Waiting times for more urgent treatments (such as a coronary bypass) were substantially lower than for less urgent treatments (such as elective hip and knee replacement, or cataract) across different OECD countries in the pre-pandemic period [24].

There is also some evidence of waiting time prioritization across patients within a given treatment. Gutacker *et al*. [25] showed that inpatient waiting times for hip replacement, from specialist addition to the list to surgery, are shorter for patients in higher pain and reduced mobility in England. Kasteridis *et al*. [26] showed, however, that there is less waiting time prioritization for outpatient waiting times, from GP referral to the specialist visit. They also showed that inpatient waiting time prioritization in England becomes more pronounced when waiting times gradually increased between 2015 and 2019, and increased further in 2020 during COVID-19. The same study showed that even more pronounced inpatient wait prioritization, from specialist addition to the list to surgery, can further reduce the total health losses from waiting [23, 26].

Successfully introducing policies that encourage further prioritization across and within treatments can be challenging. Askildsen *et al*. [27] showed that the introduction of maximum recommended waiting time in Norway that could differ by health condition and patient severity did not appear to improve prioritization. There is also some evidence on mis-prioritization of patients on the list with patients with lower socioeconomic status waiting longer for publicly-funded care than patients with higher socioeconomic status (see for example [28] for England [29, 30]; for Norway; and [31, 32] for Australia).

### Waiting times and labour market outcomes

For individuals in the working age, prolonged waiting times can also have negative consequences on employment or other labour market outcomes. Those already employed might end up on sick leave and claim sickness benefits if their health condition prevents them from performing their usual tasks [7]. Even if they continue to work, their productivity may be adversely affected. Individuals looking for a job may find it harder to find employment or will have to limit their job search to less demanding roles [8]. In the most severe cases, individuals may become economically inactive and give up looking for employment. Long waiting times for health care can create a negative spill-over from the health sector to the labour market.

There is a limited but growing literature that documents the effect of longer waiting times on labour market outcomes. Godøy *et al*. [7] showed that in Norway long waiting times for orthopaedic surgery have persistent reductions in labour supply through an increase in work absences and permanent disability receipt. The effect appears to be substantive. An increase in waiting times by 10 days increased health-related work absences over the 5 years following referral by 8.7 days. It also increased the probability of a patient entering a permanent disability program by 0.4 percentage points by the end of fifth year. The effect was concentrated amongst patients who were already on sick leave at the time of referral. For these patients, an increase in waiting times by 10 days increased health-related work absences by 27.2 days over 5 years, and increased disability rate by 1.3 percentage points. Instead, there was no effect for patients who were working at the time of the referral. This suggests that waiting times create barriers to returning to work for patients who are already on sick leave.

Prudon [8] found that in the Netherlands an increase in waiting time of 1 month for mental health services in the Netherlands reduced the probability of being employed by two percentage points and increased the probability of receiving sickness or disability benefits by one percentage point. The effect was larger for patients with lower educational attainment.

Dodd *et al*. [33] also focused on mental health services that were provided by the NHS Talking Therapies programme in England. They found that an increase in waiting times (measured at area level) by 10 days increased the gap in the probability of employment between those who are in good and poor mental health by 1.5 percentage points, and the probability of taking time away from work by one percentage point.

Warner and Zaranko [34] investigated the extent to which the increase in health-related benefit claims in England (about 40% between 2018 and 2024) can be explained by waiting lists and waiting times. They found that at the local level there is no clear relationship between changes in NHS waiting times and health-related benefit claims over time. The authors cautiously suggested that in some cases there is instead some evidence suggestive of a weak positive relation between waiting and benefit claims for mental health conditions and orthopaedic care, which is in line with the evidence presented above. One limitation of the analysis is that waiting times were measured for the whole population, as opposed to working age population.

### Health and labour market outcomes

Although there is a limited literature specifically looking at waiting times for health services on labour market outcomes, there is a vast literature documenting the effect of health or health care on labour market outcomes. The recent review by Pintor *et al*. [11] provided extensive evidence on the effect of health on earnings and labour supply, measured by employment rates and increased hours of work. Out of 110 studies, 67% of the labour market outcomes showed that better health had a positive effect on labour market outcomes (or, conversely, ill health had a negative effect on labour market outcomes), 12% showed no effect, and 21% showed a negative effect. The findings covered both low-, middle-, and high-income countries and were robust to a range of health indicators and conditions, such as self-reported health, chronic diseases, nutritional health, infections, and mental health.

There is also evidence that improved access to health care affects labour market outcomes. For example, del Valle [35] showed that public health insurance expansion in Mexico increased labour supply of informal workers. Goodman-Bacon [36] showed that the childhood Medicaid programme in the US had positive long-term effects on employment and reduced receipt of disability transfer programmes up to 50 years later, in addition to better health. Brown *et al*. [37] further showed that the federal government recovered 58 cents for each dollar invested in childhood Medicaid.

## Discussion

The review of the evidence suggests that waiting times for non-emergency care generate health losses. The main loss appears from the postponement of health benefits, with a smaller role played by reductions in the ability to benefit, which varies by health conditions. There is evidence that longer waiting times affect labour market outcomes, and this appears to be the case in particular for mental health services and orthopaedic care. We therefore discuss possible areas for policy actions and interventions in these two areas, where reductions in waiting times could generate positive co-benefits for labour market outcomes.

For mental health services, even before COVID-19, there was a recognition that care in this area has been historically underfunded across several high-income countries [1]. These concerns are backed up by the evidence above that shows that long waiting time for mental health care can increase mortality [18] or lead to mental health deterioration [19]. Timely access to mental health services can help individuals to return to work quickly: the longer individuals are on sick leave, the less likely they are to return to work in the medium to long term. Early interventions also require coordination of mental health professionals with primary and secondary care providers, but these can be difficult to set up due to heavy workloads in each of the segments of the health system and lack of appropriate financial arrangements.

The policy focus on mental health has been aimed at better meeting demand through increased service volumes or scope, rather than managing demand [1]. The need for action is reflected in the Final Report of the Commission for Healthier Working Lives by the Health Foundation [38] that emphasizes the need for early interventions to encourage people to stay in work. There are, however, obstacles in mobilizing resources for mental health given by pressure on public budgets, difficulty in recruiting and retaining health workers, and diverting health care resources from physical to mental health.

The evidence also highlights a second area of intervention that relates to orthopaedic care. There is evidence that long waiting times for orthopaedic care can prolong the duration of time during which people are on sick leave. Given that many patients are not at immediate risk of health deterioration, patients tend to have very long waiting times for this type of care. The evidence suggests that orthopaedic treatments can be highly effective in reducing pain and let patients regain mobility [21, 22]. The very long wait therefore generates a substantive health loss and, in addition, prevents people from going back to work [7], especially for those workers whose jobs are physically demanding.

One policy option for orthopaedic care would be to prioritize such patients relative to other areas of health care. However, this may be at the cost of increasing waiting times for care that is more urgent and where delays could affect health outcomes, as for cardiovascular conditions. An alternative would be to further prioritize patients on the list for orthopaedic patients by reducing the waiting time for patients who are less mobile or in higher pain and therefore at risk of having to stop working. Improving prioritization across patients could minimize the total health loss from waiting [26], while reducing the risk of going on sick leave and suspend working. This could be combined with interventions by employers to adapt the tasks to individual health circumstances and prevent them from stopping working for longer periods which makes it less likely that individuals will return to work in the medium to long term. As waiting times increase, some patients resort to privately funded care. But only a few patients can afford it and even the private sector can face capacity constraints as both the public and private sector draw from the same health workforce. Improving access to health care and reducing delays require careful management of demand to reduce and eliminate inappropriate referrals. It also requires increasing the productivity of health workers and expand capacity, while attracting and retaining health workers through good working conditions and appropriate remuneration.

Although the discussion of prioritization has focused on orthopaedic care, for which more links with labour market outcomes have been established, similar issues may arise in other areas of health care. Reducing delays across the health systems will also require similar interventions. Expanding capacity, however, will require mobilizing more resources for the health sector. This can be challenging given increasing pressures on public finances. To make the case for investing in health, it is important to document not only the returns in terms of health, but also the co-benefits that arise more broadly from health to labour market outcomes [9–11].

## Conclusion

In addition to generating health losses to patients, long waiting times can have adverse effects on labour market outcomes for individuals in the working age through reduced productivity, longer sick leave, or employment. Enhanced prioritization of patients on the list and re-allocation of resources within the health sector and across sectors could mitigate such effects.

Conflict of interest: None declared.

## Data Availability

All data used in the manuscript are publicly available from the sources provided in the study.
